# Coronary Revascularization Using Bilateral Internal Thoracic Arteries: Safe with Skeletonization?

**DOI:** 10.4172/2155-9880.S7-007

**Published:** 2013-11-20

**Authors:** Brody Wehman, Bradley Taylor

**Affiliations:** Division of Cardiac Surgery, University of Maryland Medical Center, Baltimore, MD, USA

**Keywords:** Bilateral internal thoracic artery, Coronary revascularization, Skeletonization sternal wound infection

## Abstract

Substantial evidence exists to support a long-term survival benefit with bilateral internal thoracic artery (BITA) revascularization in coronary artery bypass grafting. However, this technique remains grossly underutilized worldwide and especially in the United States. In this review, we discuss evidence for the advantages of BITA grafting as well as the associated the risk of sternal wound complications. We then review a growing body of literature that suggests ‘skeletonization’ of the internal thoracic artery during harvest confers a protective benefit against sternal wound infection in patients receiving BITA.

## Introduction

The left internal thoracic artery (LITA) is well established as the conduit of choice in coronary artery bypass grafting (CABG), with clear advantages over saphenous vein grafts. However, use of the right internal thoracic artery (RITA) in addition to LITA, or bilateral ITA (BITA) grafting, is used in only 4% of cases in the United States [1], in spite of substantial evidence that BITA provides a long-term survival advantage. Much of the resistance to BITA usage centers on concern for an increased risk of sternal wound complications, especially in diabetic patients.

In this review, we discuss evidence for the advantages of BITA grafting as well as the associated the risk of sternal wound complications. We also review increasing evidence that skeletonization of the ITA confers a protective benefit against sternal wound infection in patients receiving BITA.

## ITA Use in CABG Surgery: Distinct Advantages

The ITA has been long established as the optimal conduit for coronary artery bypass grafting, offering superior long-term patency, freedom from re-intervention and survival rates [2–4]. This is largely attributed to the unique physiologic properties of the ITA [5,6] (Table 1).

Additionally, these clinical and physiologic benefits are complemented by the highly practical anatomic location of the ITA. Coursing parallel to and 3 to 5 centimeters lateral to the sternum, the vessel is readily exposed in routine patients undergoing median sternotomy for CABG.

## Use of Bilateral Internal Thoracic Arteries

### Survival benefit

Among the earliest to describe use of BITA in CABG was Rene Favaloro while at the Cleveland Clinic in the late 1960’s [7]. Pioneering surgeons from Cleveland Clinic and elsewhere continued to use BITA, reporting excellent results through the 1980’s and 1990’s [8,9]. In a landmark paper from Lytle et al in 1999, “Two Internal Thoracic Artery Grafts are Better Than One”, the authors demonstrated superior freedom from re-operation and overall survival in patients receiving BITA versus SITA, with 5, 10 and 15 year survival rates of 94%, 84% and 67% for the BITA group and 92%, 79%, and 64% for the SITA group, respectively (p<0.001) [9]. Although this study was a retrospective, single-center review it included over 10,000 patients and used propensity matched scoring to compare those receiving SITA (n=8,123) versus BITA (n=2001) during CABG. It was the largest study to date providing evidence for a survival benefit with BITA grafting. Large retrospective studies have continued to demonstrate significantly improved survival over 20 and 30-year follow-up periods for patients receiving BITA versus SITA grafting [10,11].

Yet broad conclusions on the survival benefit of BITA remain limited by lack of data from randomized, prospective studies. Accordingly, current ACC/AHA Guidelines list BITA grafting as a Class IIA recommendation, with Level of Evidence B, which signifies the recommendation is “based on evidence from a single randomized trial or nonrandomized studies” [12]. A systematic review in 2001 by Taggart et al. identified only 9 cohort studies and no randomized trials [13]. While this meta-analysis demonstrated superior survival in those receiving BITA, the authors acknowledged the limited nature of their findings, highlighting the need for a large randomized trial, which Dr. Taggart initiated soon thereafter. This trial, known as the Arterial Revascularization Trial (ART), is a multi-center, randomized control trial comparing bilateral versus single ITA grafting with a primary outcome of survival at 10 years. In order to detect a 5% reduction in 10 year mortality, and remain adequately powered (90%) at a 5% significance level, the trial requires enrollment of approximately 3,000 patients. Recently, the one-year results were published and while no survival benefit was detected, long-term follow-up remains ongoing. There was an increased incidence of DSWI in BITA compared to SITA (1.9% vs. 0.6%, respectively), and approximately half of these occurred in diabetic patients [14].

### BITA use and risk of deep sternal wound infection

Although existing long-term data point toward a survival benefit, the risk of DSWI remains a primary source of concern for cardiac surgeons considering BITA revascularization, particularly in diabetic patients. The association between wound complications and BITA use was initially reported in small series throughout the 1970’s and 1980’s, occurring in 1.5% to 4.0% of non-diabetic patients, and in 5.7% of diabetic patients [15–19]. A large retrospective study from Loop et al in 1990 observed that diabetic patients receiving BITA were 5 times as likely to suffer wound complications [20]. Borger et al further demonstrated the risks associated with BITA revascularization, focusing specifically on the incidence of DSWI. In a review of over 12,000 patients, they reported the risk of DSWI in diabetic patients increased from 1.3% to 14.3% when using BITA grafting (p=0.001, odds ratio 3.2) [21]. Furthermore, they compared male diabetic versus non-diabetic patients undergoing BITA and found 20% of male diabetic patients who received BITA suffered a DSWI vs. 1.6% of male diabetic patients receiving SITA. In a recent review of the Nationwide Inpatient Sample, Itagaki et al reviewed 1,526,360 patients who underwent CABG and compared BITA versus SITA use and development of DSWI [22]. They found that BITA usage was associated with a DSWI rate of 1.4%, and the presence of severe, chronic diabetes was a significant risk factor for DSWI (OR 1.57). However, the investigators reported that BITA use alone was not an independent predictor of DSWI (OR 1.03). Additionally, another recent review found no difference between DSWI among matched groups of SITA vs. BITA (7 of 414 [1.7%] versus 13 of 414 [3.1%]; P=0.179) and that, interestingly, the previously discussed survival benefit from BITA grafting extended to diabetic patients (median survival: SITA, 9.8 years versus BITA, 13.1 years; P=0.001) [23]. Yet while some recent reports are encouraging, the historically reported incidence of DSWI for patients receiving BITA ranges from 1.2 to 2.4%, and from 14 to 20% in diabetic patients. This is in stark contrast to the established incidence of DSWI following CABG with SITA grafting, which ranges from 0.49 to 1.6% of patients [24].

The increased incidence of DSWI observed in BITA grafting is directly related to reduced blood flow to the sternum. This combined with an aging population with the inherent risk factors associated with coronary artery disease results in a poor environment for sternal wound healing, setting the stage for sternal necrosis, dehiscence and infection. Experimental work in large animals has quantified the degree to which ITA harvest diminishes the sternal circulation [25,26]. In primates, for example, after injecting microspheres labeled with radioactive isotopes into each ITA investigators were able to accurately assess sternal blood flow using gamma counting. While sternotomy alone had no effect on sternal circulation, blood flow to the sternal halves in which the IMA was harvested decreased dramatically (from 4.5 to 0.8 ml/gm/min; p < 0.001) [25]. To highlight the importance of the ITA and its collateral blood supply to sternal circulation, one study injected contrast into the ITAs of 50 human cadaveric anterior chest walls [27]. They were then able to meticulously identify and dissect the ITA and every collateral branch. In doing so, they identified four anatomical subtypes based on the branching patterns of collateral blood vessels from the ITA (Table 2).

In some types, the collateral circulation would not appear to be at risk with ITA harvest; in other subtypes, due to the location of branching from the ITA, the collateral supply is at high risk for disruption. The anatomic descriptions from this work underscore the importance of making every effort to preserve the collateral circulation during ITA dissection and clip application. They also showed a decreasing number of collateral branches in the intercostal spaces from cephalad to caudad, which suggests the inferior sternum is less collateralized and at a higher risk for sternal wound complications.

## Use of Skeletonized BITA Reduces DSWI

### Technique of pedicled versus skeletonized ITA harvest

Conventional ITA harvest involves dissection of a pedicled graft from the chest wall, which includes the ITA, internal thoracic vein, lymphatics, fat and surrounding tissue. This method is safe, efficient and reproducible in CABG with SITA. However, due to sacrifice of venous drainage and often disruption of collateral vessels, the use of this technique in BITA revascularization may be the greatest contributor to the observed increase in DSWI. An alternative method of dissecting the ITA from the chest wall, known as ‘skeletonization’, involves dissecting free only the artery from the endothoracic fascia, thereby maintaining the surrounding venous, lymphatic and collateral blood supply. Additionally, proponents of skeletonization frequently employ a “scissor only” dissection technique to avoid electrocautery-induced thermal injury to the ITA and collateral vessels [28]. Theoretically, skeletonization of the ITA leaves enough of the sternal circulation intact to facilitate proper wound healing [29,30]. A growing body of evidence suggests that DSWI rates in BITA are reduced to that of SITA revascularization, even in diabetics, when the ITA is skeletonized.

### Clinical studies

Several observational studies have reported safety of BITA grafting with skeletonization. Bical et al [28] reviewed their experience with 560 consecutive patients receiving skeletonized BITAs. Remarkably, they reported sternal complications in only 6 (1.1%) patients and 0 wound complications in 63 diabetic patients receiving skeletonized BITA grafts. However, this study was limited by lack of a comparison group. Calafiore et al [31] compared skeletonized versus pedicled BITA by era. In the early group, all ITA were pedicled versus the later group in which all ITA were skeletonized. The investigators found a 10% incidence of sternal wound complications in BITA diabetic patients receiving pedicled grafts vs a 2.2% incidence in BITA diabetic patients receiving skeletonized grafts (p < .05). Despite these impressive findings, the study design lacked a matched comparison of skeletonized versus pedicled BITA grafting. Peterson and Borger et al, whose previous work demonstrated striking evidence for the increased risk of DSWI with BITA use [21], sought to specifically examine the risk of DSWI in diabetic patients undergoing skeletonized versus pedicled ITA harvest [32]. There were 79 diabetics patients who received skeletonized and 26 matched diabetic patients who received pedicled ITAs included in the analysis. They found that DSWI was significantly lower in the skeletonized group (1.3% vs 11.1%, p=.03), as was any sternal wound infection (superficial or deep) (5.1% vs 22.2%, p=.03). Average total operative time was slightly longer in the skeletonized BITA group, but this was not statistically significant (199.3 vs. 184.7 minutes, p=.3). Importantly, when compared to non-diabetic patients who underwent conventional, pedicled BITA (n=578), the investigators found no difference (1.2% vs 1.6%). The authors concluded that as long as the ITAs were skeletonized, diabetes was no longer a contraindication to BITA grafting. A similar study with a larger group of BITA patients (pedicled=300, skeletonized=150) found that when BITA were skeletonized, there was no difference in DSWI between diabetic and non-diabetic patients [33]. Additionally, they reported that skeletonization in BITA patients had an equivalent DSWI incidence as those with pedicled SITA.

### Laboratory data

Dr. Frank Spencer’s group from NYU found a significant reduction of sternal blood flow in dogs that underwent a pedicled versus skeletonized ITA harvest [34]. In this study, 8 dogs underwent BITA, with one ITA harvested as a pedicle and the contralateral vessel skeletonized. Remaining blood flow to the chest wall was then measured using radioactive microspheres and a gamma counter. Blood flow to the sternal halves where the ITA had been skeletonized was significantly greater than flow to sternal halves where a pedicled graft had been harvested (2.60 +/− 0.68 versus 1.27 +/− 0.27 cm3/min/100 gm, p < 0.001). These data provide some physiologic evidence for the protective effect of skeletonization.

### Results from meta-analyses

Over the last decade several extensive meta-analyses have examined skeletonization in BITA and DSWI [35–38]. Saso et al included 12 studies for review and reported a reduction in the odds of sternal wound infection in all BITA patients receiving skeletonization (OR=.41), with an even greater reduction in diabetic patients receiving BITA (OR=.19) [35]. Another group recently reviewed 10 observational studies and 1 randomized trial to analyze a pooled total of 126,000 diabetic patients, 122,500 receiving SITA and 3800 BITA [37]. The authors found the risk ratio for DSWI in BITA versus SITA in all diabetic patients was 1.71. However, in a sub-analysis of BITA patients who underwent skeletonized harvest, there was no difference in the risk of DSWI in SITA patients. Pedicled ITA had an increased risk ratio of 1.77. Taken together, the data compiled from these meta-analyses provide us with an estimated incidence of DSWI in all patients receiving skeletonized BITA grafts of 1.1 to 1.7%, while the incidence in diabetic patients ranges from 1.2 to 2.2%.

### Emerging strategies to minimize DSWI

The advent of robotic totally endoscopic coronary artery bypass (TECAB) and minimally invasive coronary artery bypass (MIDCAB) grafting provides access to the ITA and heart without sternal division. Accordingly, the incidence of DSWI and mediastinitis is virtually non-existent in published TECAB and MIDCAB literature [39]. The increased magnification and dexterity afforded by the telerobotic system allows for safe and efficient skeletonization of the ITA. In utilizing these minimally invasive strategies, patients are able to enjoy the survival benefit of bilateral arterial revascularization without the associated risk of sternal wound complications.

## Conclusion

Although data from randomized trials are lacking, there is substantial evidence from observational studies that BITA revascularization provides a significant survival benefit in patients undergoing CABG. And while several observational studies and meta-analyses report BITA use to be safe in diabetic patients with the use of ITA skeletonization, widespread adoption of BITA grafting as a revascularization strategy will likely not occur until further evidence is obtained from randomized controlled trials. Finally, the application of minimally invasive, sternal-sparing approaches to CABG may help facilitate increased and safer use of BITA revascularization.

## Figures and Tables

**Table 1:** Physiology of the internal thoracic artery.

Absent or very thin vasa vasorum
Dense internal elastic lamina without fenestrations
High integrity of endothelium
Thin medial layer with few smooth muscle cells
Enhanced secretion of prostacyclin and nitric oxide

**Table 2:** Morophologic variants of sternal blood supply.

Type 1	Sternocostal branch with both sternal and perforating artery communicating with anterior intercostal artery
Type 2	Sternocostal branch with perforating and anterior intercostal artery arising from common artery
Type 3	Sternal-perforating branch not connected to the anterior intercostal artery
Type 4	All sternal branches arise as separate arteries from common artery
